# First case report of disseminated gonococcal arthritis in Newfoundland and Labrador

**DOI:** 10.1099/acmi.0.000807.v3

**Published:** 2025-01-30

**Authors:** Nessika Karsenti, Matthew Nelder, Jennifer LeMeissurier, Samuel Bourassa-Blanchette, Robert M. Taylor

**Affiliations:** 1 Faculty of Medicine, Memorial University, St. John’s, NL, Canada; 2 Newfoundland and Labrador Health Services, St. John's, NL, Canada

**Keywords:** DGI, *N. gonorrhoeae*, septic arthritis, stigmatization

## Abstract

**Introduction.** Disseminated gonococcal infections (DGIs) are a rare but often debilitating complication of *Neisseria gonorrhoeae* infections. Often presenting as arthritis–dermatitis syndrome, true suppurative joint infections are an even more rare form of DGI. Here, we present the first known case of DGI in Newfoundland and Labrador in over 10 years.

**Case report.** A 50-year-old man who is known to inject drugs with multiple housing and social challenges presented to the emergency department with a 2-day history of an isolated, painful, erythematous knee. After being assessed by orthopaedics and undergoing an operative debridement, intraoperative cultures grew *N. gonorrhoeae*. He was treated with intravenous ceftriaxone, and his course in hospital was complicated by inadequate pain control and a lack of stable housing.

**Conclusion.** Although rare, DGIs need to remain on every clinician’s differential for septic arthritis, given the increasing prevalence of gonorrhoeal infections in Canada, particularly in Newfoundland and Labrador. In addition, patients who are at risk of delaying accessing care, such as people who inject drugs and unhoused individuals, are at higher risk of complicated hospital stays.

## Data Summary

All data associated with this work are reported within the article.

## Introduction

Gonorrhoea is a sexually transmitted infection caused by the fastidious bacterium *Neisseria gonorrhoeae*. The incidence of gonorrhoea continues to increase in countries across North America, the Western Pacific and Europe [1]. Newfoundland and Labrador is not immune, observing a fivefold increase in cases from 2019 to 2022 (10.1–49.2 cases per 100 000 persons) [2,3]. The highest incidence of gonorrhoeal infections in Canada continues to be amongst males aged 25–29 years [2]. Clinically, gonorrhoea presents in a variety of manifestations, most commonly as a urogenital disease. Disseminated gonococcal infections (DGIs), which extend beyond urogenital symptoms, have increased in prevalence alongside rising urogenital rates but still only represent 0.5–2% of all cases [4,5]. In Canada, a significant rise in the incidence of DGIs has been observed, increasing from 0.03 to 0.20% of all reported *N. gonorrhoeae* infections between the years of 2016 and 2020 [6]. Among DGIs, less than 50% of cases present as true arthritis [7]. Most cases of DGI present as a triad with migratory painless arthralgia (70% of cases), tenosynovitis (67% of cases) and a painless, pustular dermatitis (67% of cases) [8]. This triad is also called arthritis–dermatitis syndrome and generally appears 2–3 weeks after infection. Most cases (63%) also present with fever in the acute phase of illness [8]. Only 32% of cases present as acute purulent mono-arthritis [8]. *N. gonorrhoeae* is the most common causative organism of monomicrobial septic arthritis in sexually active adults [9].

## Case presentation

A 50-year-old man with a known cocaine intravenous (IV) drug use history, poorly controlled diabetes and multiple social and housing challenges presented to the emergency room with a 2-day history of a progressively swollen, painful left knee. The patient denied persistent fevers, other infectious symptoms or a previous history of trauma in the knee. He denied recent penile discharge or dysuria. Importantly, he endorsed injecting cocaine one to four times a day, often in his lower anterior legs. While he had a documented history of opioid injection as well, he denied the use of any drugs other than cocaine at the time of presentation and was compliant with his 8 mg of suboxone. He reports monogamy with one heterosexual partner but does not use barrier protection.

At the time of presentation, he was unable to bear weight, and his range of motion in the affected knee was markedly decreased. Track marks were evident on his bilateral arms and lower legs, and he also had a dry, well-healing ulcer on the dorsum of his right foot. Otherwise, he had no signs of dermatitis, tenosynovitis or pain beyond the affected knee and was afebrile. Preliminary blood analysis revealed a white blood cell count of 143 000 000 cells per litre and a C-reactive protein (CRP) level of 20 mg l^−1^. He was evaluated by orthopaedic surgery, diagnosed with septic arthritis, given a one-time dose of 1.5 g of IV vancomycin and underwent a left knee arthroscopy and irrigation with debridement the same day.

**Fig. 1.**
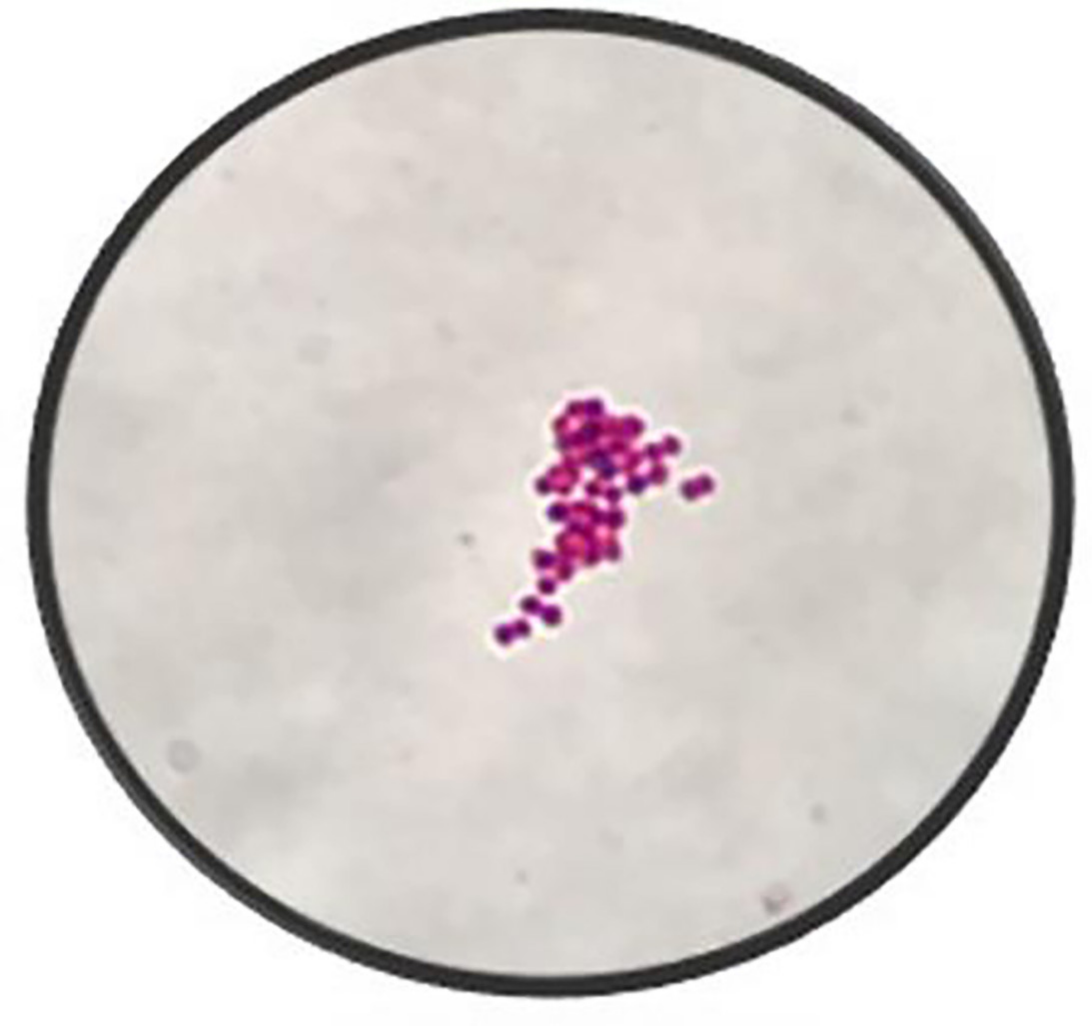
Gram stain of synovial fluid under 100× oil immersion.

**Fig. 2.**
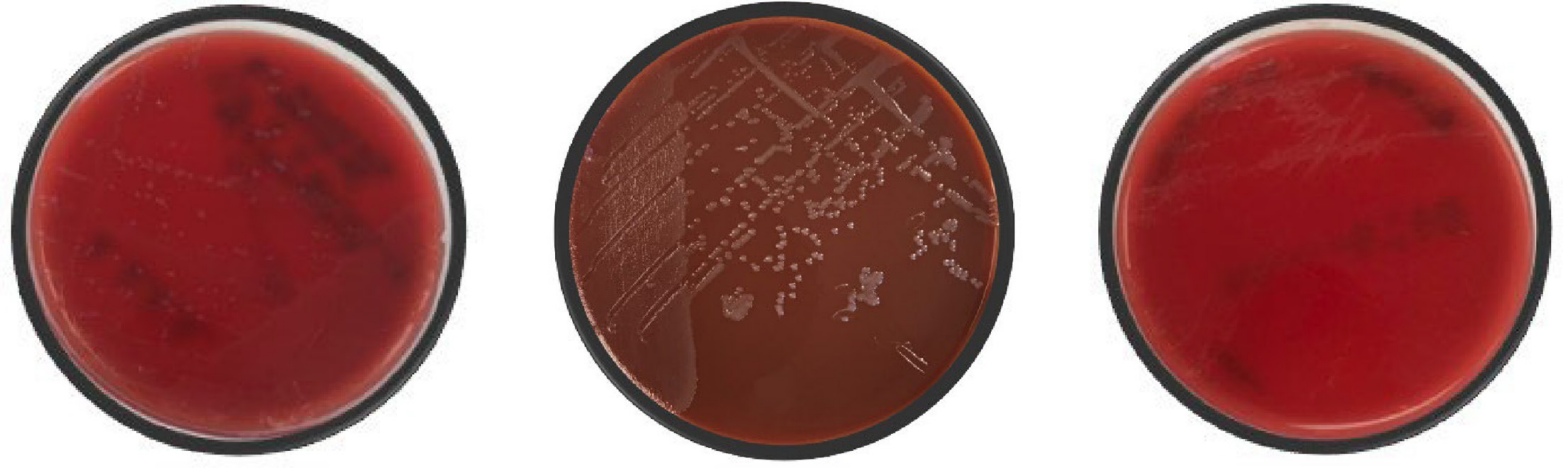
Culture growth of *N. gonorrhoeae* on bacterial medium. Left to right, Tryptone soya agar with 5% Sheep Blood Oxoid (TSA, primary inoculation), Chocolate Agar Oxoid (primary inoculation) and TSA (subculture from the primary TSA plate).

Synovial fluid collected from the operating room demonstrated a cell count of 78 000 cells per litre and 93% neutrophils. On post-operative day 1, his Gram stain results (Fig. 1) showed Gram-negative diplococci, and infectious diseases were consulted. The differential diagnosis was narrowed to either gonococcal or meningococcal infection based on his Gram stain results, and the recommendation was made to change his antibiotic regimen to 2 g of ceftriaxone once daily. In addition, given the disseminated nature of his infection, the patient was further worked up for immunodeficiency states and other sexually transmitted infections and underwent both rectal and throat swabs for gonorrhoea and chlamydia to identify a source. Two days later, on post-operative day 3, his culture results further narrowed the pathogen to *N. gonorrhoeae* (Fig. 2), which was confirmed by the Bruker Biotyper MALDI-TOF MS instrument (Billerica, MA, USA).

**Fig. 3.**
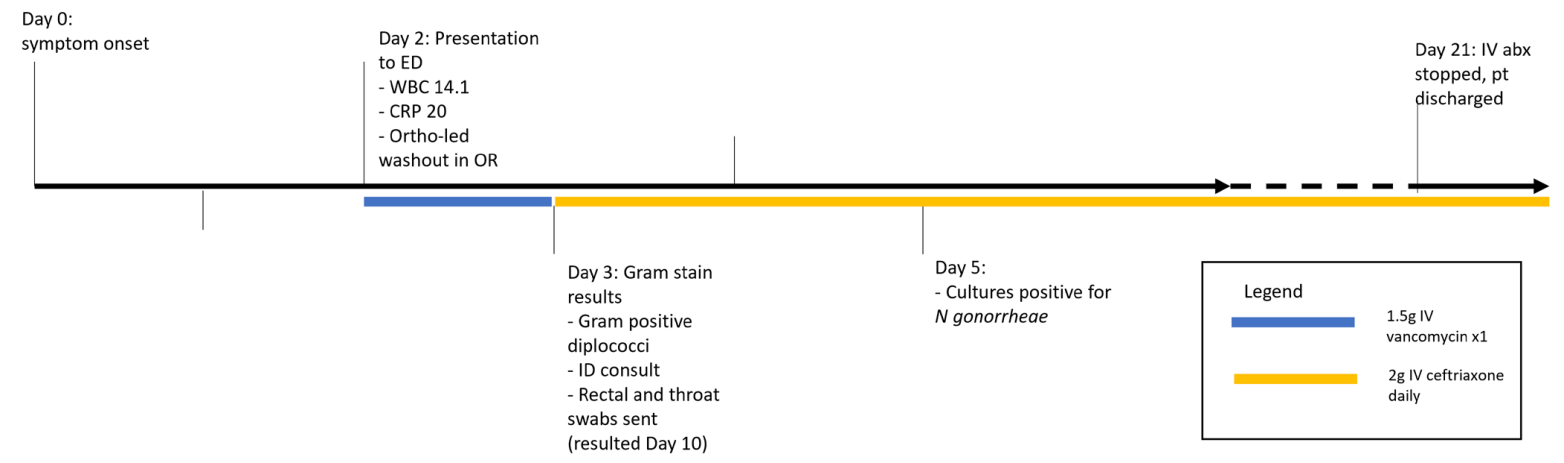
Timeline of events and investigations.

Further workup showed a negative rectal swab but positive throat swab for *N. gonorrhoeae*. His blood and urine cultures were negative. He had normal complement and immunoglobulin levels; the patient was also HIV and hepatitis B negative but was found to be hepatitis C virus (HCV) positive. Given he denied needle sharing and was followed closely by the harm reduction clinic (where he obtained his suboxone), he was felt to be at low risk for HIV and thus was not started on post-exposure prophylaxis [10]. Treatment for HCV was initiated with the help of social work colleagues in obtaining funding. The initial recommendation for infectious diseases was for a total of 7–14 days of antibiotics, with a step-down from IV ceftriaxone to oral cefixime when clinical improvement was noted; these agents were selected based on Canada’s local antibiotic resistance patterns. The patient ultimately received a total of 19 days of IV ceftriaxone and progressed steadily with physiotherapy; his surgical site healed well, and he was discharged from the hospital with social support and follow-up for his HCV. Prior to discharge, the patient was contacted and consent was obtained. The timeline of events is outlined in Fig. 3.

## Discussion

This case describes the only known case of DGI in Newfoundland and Labrador in at least 10 years. Newfoundland and Labrador have historically low gonococcal infection rates compared to Canada; however, rates are continually on the rise, leading to increased potential of DGIs [2]. DGIs are a uniquely concerning complication of gonorrhoeal infections, given how easily the initial infection is overlooked; studies show ~40% of men and women are found to be infected with gonorrhoea without symptoms [11,12]. In addition, DGIs are rarely part of an initial differential diagnosis given their rarity.

In asymptomatic patients of urogenital gonorrhoeal infection, such as in our case, testing and treatment of urogenital gonorrhoea may be delayed, giving ample time for the development of a DGI. Molecular testing on both urine and rectal samples was negative, but the throat swab was positive for the presence of *N. gonorrhoeae*. Further complicating the diagnosis, this patient presented with monoarticular purulent arthritis, one of the rarer forms of DGI. It is possible that routine (yearly) Sexually transmitted infection (STI) testing would have caught this infection before it spread to the knee.

Increasing rates of resistance to *N. gonorrhoeae* may also lead to the development of DGIs. In Canada, between 2010 and 2019, resistance rates increased for *N. gonorrhoeae* isolates for tetracycline (9%), erythromycin (7%) and ciprofloxacin (21%) [13]. In our case, phenotypic testing demonstrated susceptibility to cefixime (0.016 µg ml^−1^), ceftriaxone (0.008 µg ml^−1^), ciprofloxacin (0.004 µg ml^−1^), azithromycin (1.0 µg ml^−1^) and spectinomycin (≤32 µg ml^−1^), with resistance to tetracycline (2.0 µg ml^−1^) and intermediate to penicillin (0.50 µg ml^−1^). Given our local resistance patterns and the resistance pattern of this new isolate, although not available until after patient discharge, doxycycline was not selected as the antibiotic of choice despite more recent recommendations [14]. The *N. gonorrhoeae* multi-antigen sequence type (ST) for this isolate was ST-19875. This ST has become the most prevalent in Canada since its first identification in 2020 [6,13]. ST-19875 is correlated to increased azithromycin resistance (20% from 2010 to 2019); however, rates of resistance for DGI isolates in Canada remain low [6,13].

The World Health Organization has noted that increasing urbanization, poor detection rates and decreased condom use have enhanced the probability of increasing DGI incidence. Furthermore, there are important socioeconomic factors and related policies that put individuals like our patient in a far more vulnerable position and, thus, more likely to suffer from complications of DGI and other STIs. First, the patient in this case is a known injection drug user and was taking suboxone for opioid replacement. The effect of healthcare stigma on people who are prescribed opioid-substitution therapy is well documented and has many adverse health outcomes, including a reluctance to seek care, longer hospital stays and poorer quality of pain management [15,16]. Second, our patient was also unhoused; a study consisting of narrative interviews conducted in Ontario with unhoused individuals reported that they received poor quality care or were outright denied proper care when clinicians were aware of their housing situation [17]. Our patient was labelled a ‘drug-seeker’ and had sub-therapeutic pain treatment throughout his course in hospital, prompting additional requests for pain medication and perpetuating a cycle of stereotypes. He was also hospitalized for a total of 19 days, longer than the recommended 7–14-day suggested course of treatment [18,19], potentially due to his lack of stable housing and the need for our social workers to become involved in his care.

The stigmatization of an already vulnerable demographic perpetuates poor health outcomes and may interfere with a healthcare practitioner’s ability to perform their duties to the optimal level [20]. Awareness of these issues and how they impact patients is a paramount component of providing care to individuals with DGIs. Newfoundland and Labrador have recently implemented an initiative to address this ongoing concern, based on the ‘Housing First’ policy. This policy has been documented in the literature to improve patient care and mortality by emphasizing the need for stable housing [21].

## Conclusion

*N. gonorrhoeae* infections have been steadily increasing in Canada, leading to an increased incidence of DGIs despite their relative rarity. Here, we present the case of a young man whose gonorrhoeal infection was complicated by monoarticular septic arthritis requiring surgical washout and a prolonged course of antibiotics. This is the first case of a DGI in Newfoundland in over 10 years, and the epidemiology of the disease is discussed. While DGIs are never simple, the stigma this patient undoubtedly faced as an unhoused person who uses injected drugs was a complicating factor in his case.

## Abbreviations

CRP: C-reactive protein
DGIs: disseminated gonococcal infections
HCV: hepatitis C virus
IV: intravenous
ST: sequence type
STI: sexually transmitted infection

## References

[1] Kirkcaldy RD, Weston E, Segurado AC, Hughes G (2019). Epidemiology of gonorrhoea: a global perspective. Sex Health.

[2] Public Health Agency of Canada (2022). Report on sexually transmitted infection surveillance in canada, 2019. https://www.canada.ca/en/public-health/services/publications/diseases-conditions/report-sexually-transmitted-infection-surveillance-canada-2019.html.

[3] Newfoundland Labrador Canada (2020). Surveillance and disease reports - health and community services. https://www.gov.nl.ca/hcs/publichealth/cdc/informationandsurveillance/.

[4] Weston EJ, Heidenga BL, Farley MM, Tunali A, D’Angelo MT (2022). Surveillance for disseminated gonococcal infections, Active Bacterial Core Surveillance (ABCs)-United States, 2015-2019. Clin Infect Dis.

[5] Birrell JM, Gunathilake M, Singleton S, Williams S, Krause V (2019). Characteristics and impact of disseminated gonococcal infection in the “Top End” of Australia. Am J Trop Med Hyg.

[6] Public Health Agency of Canada (2022). Enhanced surveillance of antimicrobial-resistant gonorrhea in canada (infographic). https://www.canada.ca/en/public-health/services/publications/diseases-conditions/enhanced-surveillance-antimicrobial-resistant-gonorrhea-canada-infographic.html.

[7] Bardin T (2003). Gonococcal arthritis. Best Pract Res Clin Rheumatol.

[8] O’Brien JP, Goldenberg DL, Rice PA (1983). Disseminated gonococcal infection: a prospective analysis of 49 patients and a review of pathophysiology and immune mechanisms. Medicine.

[9] Wang DA, Tambyah PA (2015). Septic arthritis in immunocompetent and immunosuppressed hosts. Best Pract Res Clin Rheumatol.

[10] Tan DHS, Hull MW, Yoong D, Tremblay C, O’Byrne P (2017). Canadian guideline on HIV pre-exposure prophylaxis and nonoccupational postexposure prophylaxis. CMAJ.

[11] Handsfield HH, Lipman TO, Harnisch JP, Tronca E, Holmes KK (1974). Asymptomatic gonorrhea in men. Diagnosis, natural course, prevalence and significance. N Engl J Med.

[12] McCormack WM, Johnson K, Stumacher R, Donner A, Rychwalski R (1977). Clinical spectrum of gonococcal infection in women. Lancet.

[13] Sawatzky P, Martin I, Thorington R, Alexander D (2022). Disseminated gonococcal infections in Manitoba, Canada: 2013 to 2020. Sex Transm Dis.

[14] CATIE (2024). Doxycycline to help prevent bacterial STIs. https://www.catie.ca/doxycycline.

[15] Garpenhag L, Dahlman D (2021). Perceived healthcare stigma among patients in opioid substitution treatment: a qualitative study. Subst Abuse Treat Prev Policy.

[16] Cheetham A, Picco L, Barnett A, Lubman DI, Nielsen S (2022). The impact of stigma on people with opioid use disorder, opioid treatment, and policy. Subst Abuse Rehabil.

[17] Gilmer C, Buccieri K (2020). Homeless patients associate clinician bias with suboptimal care for mental illness, addictions, and chronic pain. *J Prim Care Community Health*.

[18] Li R, Hatcher JD (2023). Gonococcal arthritis.

[19] Rice PA (2005). Gonococcal arthritis (disseminated gonococcal infection). *Infect Dis Clin North Am*.

[20] Talley J (2020). The impact of social stigmas on sexual health seeking behavior: a review of literature. McNair Sch Res J.

[21] Baxter AJ, Tweed EJ, Katikireddi SV, Thomson H (2019). Effects of housing first approaches on health and well-being of adults who are homeless or at risk of homelessness: systematic review and meta-analysis of randomised controlled trials. J Epidemiol Community Health.

